# Feasibility of Replacing the Range Doppler Equation of Spaceborne Synthetic Aperture Radar Considering Atmospheric Propagation Delay with a Rational Polynomial Coefficient Model

**DOI:** 10.3390/s20020553

**Published:** 2020-01-19

**Authors:** Shasha Hou, Yuancheng Huang, Guo Zhang, Ruishan Zhao, Peng Jia

**Affiliations:** 1 School of surveying and Mapping Science and Technology, Xi’an University of Science and Technology, Xi’an 710054, China; 18210063041@stu.xust.edu.cn (S.H.); yuanchenghuang@xust.edu.cn (Y.H.); 2 State Key Laboratory of Information Engineering in Surveying Mapping and Remote Sensing, Wuhan University, Wuhan 430079, China; 3 School of Geomatics, Liaoning Technical University, Fuxin 123000, China; zhaoruishan@lntu.edu.cn; 4 China Satellite Navigation Office, Beijing 100034, China; jiap@beidou.gov.cn

**Keywords:** synthetic aperture radar, GF-3 satellite, rational polynomial coefficient, range Doppler model, atmospheric propagation delay correction, fitting accuracy, ground control point, geometric positioning accuracy

## Abstract

Usually, the rational polynomial coefficient (RPC) model of spaceborne synthetic aperture radar (SAR) is fitted by the original range Doppler (RD) model. However, the radar signal is affected by two-way atmospheric delay, which causes measurement error in the slant range term of the RD model. In this paper, two atmospheric delay correction methods are proposed for use in terrain-independent RPC fitting: single-scene SAR imaging with a unique atmospheric delay correction parameter (plan 1) and single-scene SAR imaging with spatially varying atmospheric delay correction parameters (plan 2). The feasibility of the two methods was verified by conducting fitting experiments and geometric positioning accuracy verification of the RPC model. The experiments for the GF-3 satellite were performed by using global meteorological data, a global digital elevation model, and ground control data from several regions in China. The experimental results show that it is feasible to use plan 1 or plan 2 to correct the atmospheric delay error, no matter whether in plain, mountainous, or plateau areas. Moreover, the geometric positioning accuracy of the RPC model after correcting the atmospheric delay was improved to better than 3 m. This is of great significance for the efficient and high-precision geometric processing of spaceborne SAR images.

## 1. Introduction

Since 1999, for the IKONOS satellite, considering technical confidentiality and other factors, American space imaging companies have started to provide rational polynomial coefficient (RPC) models instead of rigorous geometry models to end users as basic imaging products [1]. The RPC model is a universal geometric model of remote sensing satellite sensors, which is usually fitted by a range Doppler (RD) model in spaceborne synthetic aperture radar (SAR) imaging systems. The difference between the RPC and RD models is that the former is a purely mathematical model, is independent of the sensor, and involves simple calculations. Furthermore, with the emergence of various imaging sensors, it is difficult for end users to add new sensor models to existing software systems to process new sensor data, while the RPC model solves this difficulty well. Moreover, from the perspective of the multi-source sensor data application, the RPC model also provides a unified geometric model for the joint adjustment of multi-source, high-resolution remote sensing images. In view of these characteristics and factors, the RPC model has been widely used in photogrammetric processing of remote sensing satellite images.

Many scholars have conducted extensive research on the calculations involved in and application of RPC models. Firstly, the direct and iterative least-squares results of the RPC parameters were derived, and terrain-dependent and terrain-independent solution methods were proposed in a photogrammetric system [1,2]. Subsequently, the terrain-independent RPC solution algorithm was studied in detail, which is based on a global digital elevation model (DEM) to interpolate the maximum and minimum elevation of the study area and does not require initial values. It was noted that the third-order RPC model with unequal denominators achieved the highest replacement accuracy [3].

The replacement accuracy of the third-order RPC model was verified for different remote sensing satellite images, whether it is a high-resolution SAR satellite, such as TerraSAR-X, COSMO-SkyMed, and Radarsat-2; or medium-resolution and low-resolution SAR satellites, such as ERS, ALOS, JERS, and ASAR, it achieved accuracies of better than 0.015 pixels [3,4,5]. In addition, many scholars have used control data and compensation models (such as translation, affine transformation, and polynomial models) to improve the geometric positioning accuracies of RPC models. Consequently, the geometric positioning accuracy of the GF-3 satellite is better than 2 pixels, that of the YaoGan-18 satellite is better than 25 m, and Radarsat can achieve a positioning accuracy of 36.735 m [6,7,8,9]. 

At present, although the RPC model performs well in terms of substitution and positioning accuracy, the slant range measurement error caused by the two-way atmospheric propagation delay of the SAR signal is still not taken into account in the RPC model, and research has shown that the slant range measurement error will bring the positioning error in the range direction [10]. Meanwhile, some researchers pointed out that the range accuracy of SAR using modern satellites has been verified in centimeter-range after correcting geodynamic, systematic errors, and atmospheric effects [11,12]. Moreover, with the continuous launch of high-resolution SAR satellites, the accuracy and efficiency requirements of SAR image geometry processing have become more stringent. Therefore, in this study, we developed an RPC fitting method that corrects the atmospheric propagation delay, analyzed the feasibility of replacing the RD equation considering atmospheric delay correction with the RPC model, and verified the positioning accuracy of the RPC model of spaceborne synthetic aperture radar (SAR) considering atmospheric propagation delay.

GF-3 is the first C-band multi-polarized SAR satellite with 1 m resolution in China. It has 12 imaging modes, including SpotLight, StripMap, and ScanSAR, along with a wave imaging mode, and can provide data support services for research on water disaster monitoring and assessment, climate change, oceans, agriculture, forestry, and earthquakes. In this study, a series of experiments was conducted using GF-3 SAR satellite data and manually measured real ground control point (GCP) data from several regions in China.

Based on the RD model, this paper describes a method of fitting the RPC model of a spaceborne SAR satellite considering the atmospheric propagation delay and presents an evaluation of the feasibility of this replacement. Section 2 describes three basic models: the atmospheric propagation delay correction model, RD model, and RPC model. The RPC model fitting and accuracy verification methods that corrected the atmospheric propagation delay are presented in Section 3. The atmospheric propagation delay analysis results and RPC model fitting and positioning accuracy verification are discussed in Section 4. Finally, Section 5 presents the conclusions.

## 2. Models

### 2.1. Atmospheric Propagation Path Delay Equation

The atmospheric propagation path delay of a radar signal $\Delta L_{delay}$ depends on a complex atmospheric spatial distribution pattern, generally at a specific height $h=h_{t}$, and is represented as the integral of the sum of the dry, wet, and ionosphere components of the refractivity *N*, from $h_{t}$ to the top of the atmosphere $h_{\infty }$ [13,14]:

(1) $$N={\left(k_{1}\frac{P}{T}\right)}_{dry}+{\left(k_{2}\frac{e}{T}+k_{3}\frac{e}{T^{2}}+k_{4}W_{cloud}\right)}_{wet}+{\left(k_{5}\frac{n_{e}}{f^{2}}\right)}_{ionosphere}=N_{dry}+N_{wet}+N_{ionosphere}$$

(2) $$\Delta L_{delay}=\frac{10^{-6}}{cos\theta }\int_{h_{t}}^{h_{\infty }}\left(N_{dry}+N_{wet}+N_{ionosphere}\right)dh$$

where P is the total pressure (mbar), T is the temperature (K), e is the partial pressure of water vapor (mbar), and $W_{cloud}$ is the cloud water content (g/m^3^). These meteorological data can be downloaded from the U.S. National Center for Environmental Prediction (NCEP) website (https://rda.ucar.edu/datasets/ds083.2/). In addition, $n_{e}$ is the electronic density of the ionosphere, which can be obtained by bilinear interpolation in time and space based on the global ionospheric map provided by the European Center for Orbit Determination (CODE) (ftp://cddis.gsfc.nasa.gov/gnss/products/ionex/). Here, f and θ are the frequency and incidence angle of the radar, respectively, which can be obtained from the auxiliary file of the SAR satellite image. The constant coefficients have the following values: *k*_1_ = 77.6 K/mbar, *k*_2_ = 26.0 K/mbar, *k*_3_ = 3.75310^5^ K^2^/mbar, *k*_4_ = 1.45 m^3^/g, and *k*_5_ = 24.03310^7^ m^3^/s^2^ [15,16,17].

### 2.2. Spaceborne SAR RD Equation

The RD equation establishes the correspondence between the image and object point coordinates from the perspective of the spaceborne SAR imaging geometry. It consists of an earth model equation, SAR doppler equation, and SAR range equation [18]:

(3) $$\left\{ \begin{array}{l} \frac{X_{t}{}^{2}+Y_{t}{}^{2}}{A^{2}}+\frac{Z_{t}{}^{2}}{B^{2}}=1\\ f_{D}=-\frac{2}{\lambda R}\left(\vec{R_{s}}-\vec{R_{t}}\right)\cdot \left(\vec{V_{s}}-\vec{V_{t}}\right)\\ R=\sqrt{\left(\vec{R_{s}}-\vec{R_{t}}\right)\cdot \left(\vec{R_{s}}-\vec{R_{t}}\right)} \end{array}\right.,$$

where $R_{s}$ and $\vec{R_{t}}=\left[X_{t}Y_{t}Z_{t}\right]$ are the position vectors of the SAR satellite and target, respectively; $\vec{V_{s}}$ and $\vec{V_{t}}$ are the satellite and target velocity vectors, respectively; R is the slant range; λ is the radar wavelength; A = 6378.137 km is the mean equatorial radius; and B=(1-1/f)A is the polar radius with a flattening factor *f* = 298.255.

Considering the slant range measurement error caused by the atmospheric propagation delay, the slant range can be expressed as

(4) $$R=\sqrt{\left(\vec{R_{s}}-\vec{R_{t}}\right)\cdot \left(\vec{R_{s}}-\vec{R_{t}}\right)}+\Delta L_{delay}.$$

### 2.3. RPC Model

The RPC model uses the ratio polynomial to represent the correspondence between the ground coordinate D(Latitude,Longitude,Height) (the polar stereographic coordinate system is more applicable at 70–75 degrees latitude north or south) and the pixel coordinates d(line,sample) [19]:

(5) $$\begin{array}{l} Y=\frac{N_{l}(P,L,H)}{D_{l}(P,L,H)}\\ X=\frac{N_{s}(P,L,H)}{D_{s}(P,L,H)} \end{array}\},$$

where $N_{l}(P,L,H)$, $N_{s}(P,L,H)$, $D_{l}(P,L,H)$, and $D_{s}(P,L,H)$ are all third-order polynomials. These functions have the form

(6) $$\begin{array}{ll} F(P,L,H) & =a_{1}+a_{2}L+a_{3}P+a_{4}H+a_{5}LP+a_{6}LH+a_{7}PH+a_{8}L^{2}+a_{9}P^{2}+a_{10}H^{2}+a_{11}PLH+a_{12}L^{3}+a_{13}LP^{2}\\ & +a_{14}LH^{2}+a_{15}L^{2}P+a_{16}P^{3}+a_{17}PH^{2}+a_{18}L^{2}H+a_{19}P^{2}H+a_{20}H^{3} \end{array}$$

where $a_{i}$ is the RPC model coefficient, i=1,2,…,20; Generally, the first parameter coefficient of the denominator term is set to 1, so there are 78 parameters for RPC model.

Here, (P,L,H) and (X,Y) are the normalized ground and image coordinates, respectively. These coordinates can be expressed as

(7) $$\begin{array}{l} P=\frac{D_{lat}-D_{lat\_off}}{D_{lat\_scale}}\\ L=\frac{D_{lon}-D_{lon\_off}}{D_{lon\_scale}}\\ H=\frac{D_{hei}-D_{hei\_off}}{D_{hei\_scale}} \end{array}\}$$

(8) $$\begin{array}{l} X=\frac{s-s_{off}}{s_{scale}}\\ Y=\frac{l-l_{off}}{l_{scale}} \end{array}\},$$

where $D_{i}\left(i=lat\_off,lat\_scale,lon\_off,lon\_scale,hei\_off,hei\_scale\right)$ is the normalized parameter of the ground coordinates and $s_{j},l_{j}(j=off,scale)$ are the normalized parameters of the image coordinates.

The RPC fitting results obtained based on a variety of spaceborne SAR sensors have shown that RPC models with third-order and unequal denominators have the highest replacement accuracies [7]. The following experiments were based on this model.

## 3. RPC Model Fitting and Accuracy Verification Methods

The RPC model is essentially a mathematical model. In this study, we used a terrain-independent solution algorithm to calculate the RPC parameters. The core of the algorithm involves establishing a virtual control grid based on the SAR image, DEM data of the coverage area, and forward transformation of the RD model. Then, the RPC parameters are fitted according to the least squares fitting method and the correspondence between the ground coordinates and image pixel coordinates of the virtual control points. However, every certain virtual grid point in space is affected by atmospheric propagation delay, which causes slant range measurement error. Therefore, we used two atmospheric delay correction schemes to correct the slant range values at all virtual grid points, then re-fitted the RPC model and evaluated the fitting and positioning accuracies.

**Figure 1:**
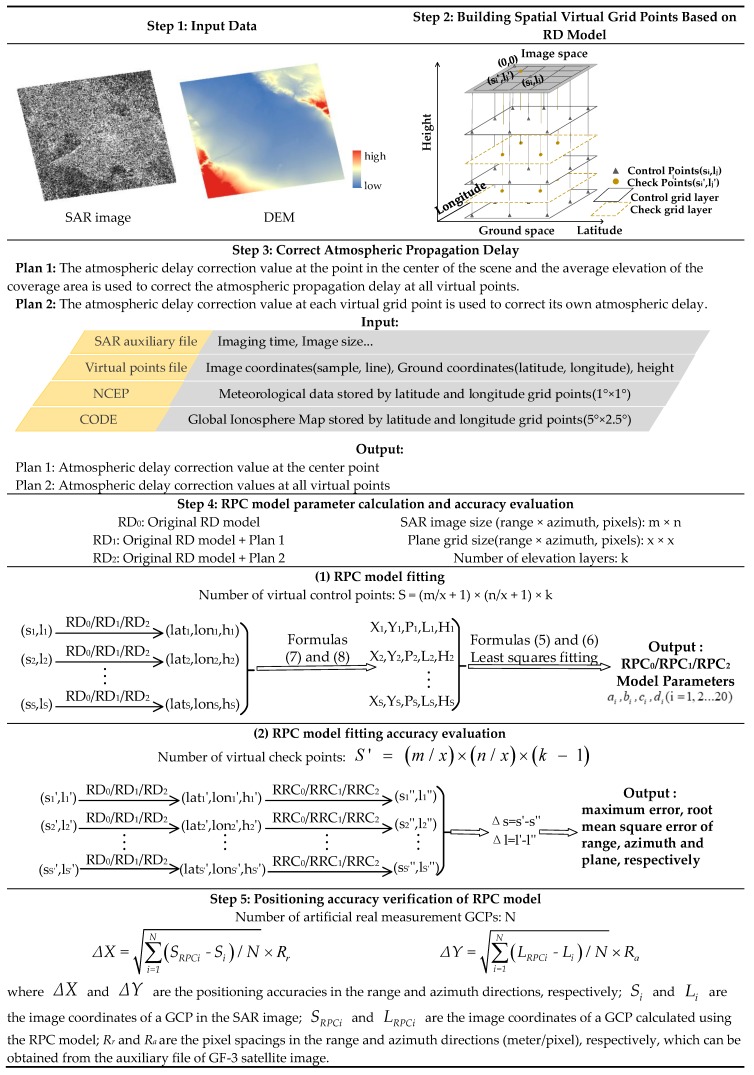
Step-by-step routine for the rational polynomial coefficient (RPC) model fitting and accuracy verification algorithm. Note: SAR = synthetic aperture radar; DEM = digital elevation model; RD = range Doppler; NCEP = National Center for Environmental Prediction; CODE = European Center for Orbit Determination; GCP = ground control point.

The steps of the algorithm are listed in Figure 1.

The steps of the algorithm can be described as follows.

**Figure 2:**
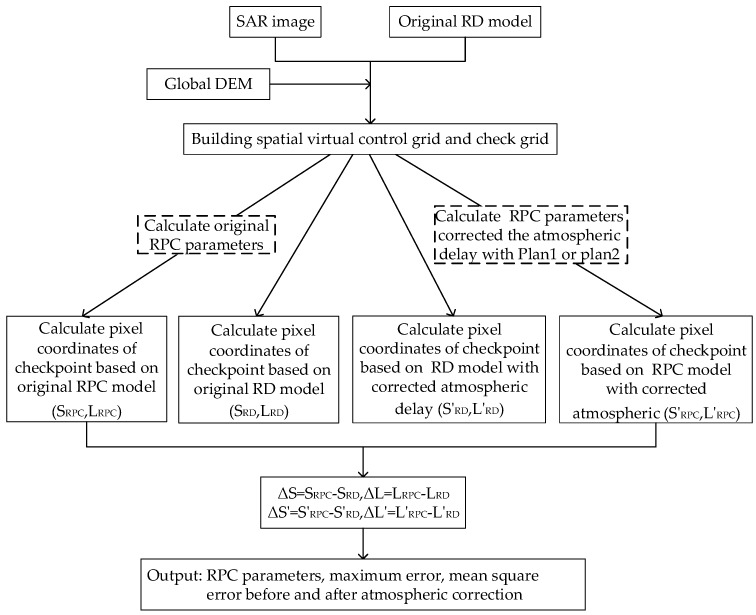
Flowchart of RPC fitting accuracy evaluation.

(1) Firstly, the input data sets, including the SAR image auxiliary file and DEM of the study area, were prepared;
(2) Then, a virtual plane grid was built according to the image size, the elevation range of the coverage area was interpolated based on the DEM data and RD model, and layering processing was performed in the elevation direction to obtain a virtual space control grid. The number of layers was required to be greater than three to prevent the design matrix from becoming ill-conditioned [1]. Next, a virtual space check grid was constructed by interpolating between the centers of four adjacent virtual control points and the two adjacent elevation layers;
(3) The atmospheric propagation delay values were corrected at all virtual grid points using two plans: one in which the atmospheric delay correction value at the point at the center of the scene and the average elevation of the coverage area were employed to correct the atmospheric propagation delay at all virtual points, and one in which the atmospheric delay correction value at each virtual grid point was used to correct its own atmospheric delay;
(4) The RPC model was fitted separately using the original RD model, RD model modified using atmospheric delay correction plan 1, and RD model modified using atmospheric delay correction plan 2, and then the fitting accuracy was evaluated. A detailed flowchart of the RPC model fitting accuracy evaluation is shown in Figure 2.

(5) The positioning accuracies of the abovementioned RPC models were evaluated based on the measured GCPs of the SAR image coverage area. To ensure the reliability of the verification accuracy, the root mean square error (RMSE) of multiple control points in one image was calculated using the equation in step 5, *∆X* and *∆Y* as an evaluation index of the geometric positioning accuracy of the RPC model.

## 4. Results and Discussion

### 4.1. Experimental Data

**Figure 3:**
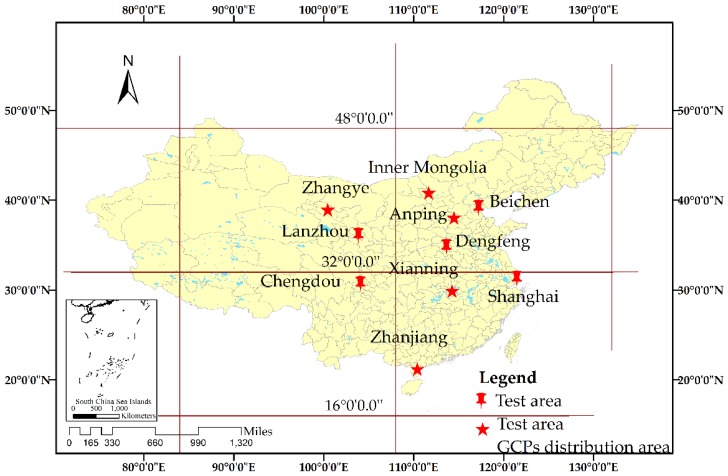
Distribution of the experimental data and GCPs.

In this study, GF-3 images of four imaging modes, namely Spot-Light (1 m resolution), Ultra-Fine-Strip (3 m resolution), Fine-Strip-I (5 m resolution), and Fine-Strip-II (10 m resolution), were used as experimental data. To verify the positioning accuracy of the RPC model considering the atmospheric propagation delay, GF-3 satellite images and GCP data from Anping, Zhanjiang, Xianning, Zhangye, and Inner Mongolia in China were used. The GCPs were derived from global navigation satellite system (GNSS) receivers, which were selected in locations with obvious features, such as the intersections of pathways. Centimeter-level positioning accuracy could be achieved using real-time kinematic static observation technology. The details of the GF-3 satellite images are provided in Table 1, and the distribution of the experimental data and GCPs is depicted in Figure 3.

### 4.2. Experimental Results and Analysis

To evaluate the feasibility of replacing the range Doppler equation of spaceborne SAR by considering the atmospheric delay with the RPC model, the following three experiments were conducted. (1) For the terrain-independent calculation algorithm of the RPC model of spaceborne SAR, the effects of the virtual grid pattern, number of elevation layers, and orbit fitting order on the fitting accuracy of the RPC model were analyzed. (2) Based on the appropriate grid pattern, number of elevation layers, and orbital fitting order, the fitting accuracy of the RPC model was assessed using the two proposed atmospheric delay correction plans. (3) Based on the fitting results of the RPC models, with and without considering the atmospheric delay of the radar signal, combined with the GCP data in the image coverage area, the positioning accuracy of the RPC model considering the atmospheric delay was verified.

#### 4.2.1. Impact of RPC Model Fitting Parameter

(1) Grid Size

**Figure 4:**
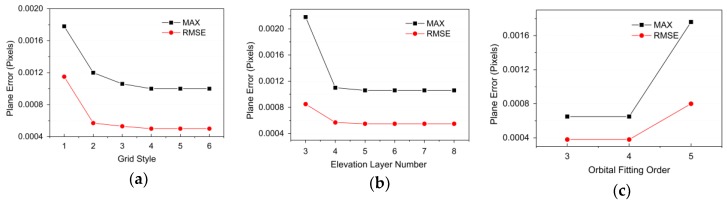
Impacts of RPC model fitting parameters: (**a**) grid size; (**b**) number of elevation layers; (**c**) orbit fitting order.

Assuming there to be five elevation layers, six different grid styles were set (style 1: 4000 pixels × 4000 pixels; style 2: 2000 pixels × 2000 pixels; style 3: 1000 pixels × 1000 pixels; style 4: 500 pixels × 500 pixels; style 5: 200 pixels × 200 pixels; style 6: 100 pixels × 100 pixels), here “N pixels × N pixels” refers to a grid spacing of N pixels, or a grid size. We take N pixels as the sampling interval in the range direction and azimuth direction, and take the sampling points as the virtual control points. Based on the fine-strip product data of the GF-3 satellite, DEM data with 30 m resolution, and the RPC fitting method described in Section 3, the influence of the grid size on the fitting accuracy of the RPC model was analyzed. The experimental results are shown in Figure 4a.

(2) Number of Elevation Layers

Using the appropriate grid pattern identified in the previous step, the number of different elevation layers (two, three, four, five, or six) was set to evaluate the influence of the number of elevation layers on the fitting accuracy of the RPC model. The experimental results are presented in Figure 4b.

(3) Orbit Fitting Order

With the appropriate grid pattern and number of elevation layers, different orders of satellite orbital fitting (third, fourth, or fifth order) were set to analyze the influence of the orbit fitting order on the fitting accuracy of the RPC model. The experimental results are depicted in Figure 4c.

Figure 4 shows that as the control grid becomes smaller and the number of elevation layers increases, the plane RMSE of the checkpoint gradually decreases. Eventually, the accuracy of the RPC model becomes close to that of the rigorous imaging geometry model. As the orbital parameter fitting order increases, the plane RMSE of the checkpoint gradually increases. To balance the fitting accuracy and computational efficiency of the RPC model, we set the grid size to 500 pixels × 500 pixels, the number of elevation layers to five, and the orbit fitting order to third-order for the subsequent study.

#### 4.2.2. RPC Model Fitting Accuracy Evaluation

Based on the atmospheric propagation delay correction model described in this paper combined with the meteorological data stored by the NCEP every 6 h, the global ionospheric map provided by CODE every 1 h, and the average elevation and size of the image coverage area provided by the GF-3 image auxiliary file, the atmospheric propagation delay correction of the radar signal was calculated to analyze the influence of atmospheric propagation delay correction on the RPC fitting accuracy.

In this study, atmospheric propagation delay correction of the radar signal was performed using two methods: one with a GF-3 image of a single scene and unique atmospheric delay correction parameters (plan 1) and one with an image of a single scene and spatially varying atmospheric delay correction parameters (plan 2). The two plans can be described as follows. In plan 1, the atmospheric delay correction at the point at the center of the scene and the average elevation of the coverage area are calculated to correct the atmospheric propagation delay at all virtual grid points. In plan 2, the atmospheric propagation delay correction value is calculated at each virtual grid point to correct its own atmospheric delay. The delay results are presented in Table 2.

In Table 2, the calculation results for plan 1 show that the maximum slant range correction value at the center point among the different images is −3.795 m, and the delay error in the ground range direction was calculated based on the incident angle of the image as approximately −5 m, which indicates that the atmospheric delay error has a significant influence on the positioning accuracy. The calculation results for plan 2 show that the atmospheric delay correction values of the virtual grid points with different spatial distributions on a scene image are different, and the slant range correction values of the virtual grid points in all of the image scenes are between 1.521 m and −3.919 m. Thus, the atmospheric delay correction values differ between imaging angles and imaging regions. It was, therefore, concluded that the atmospheric propagation delay is mainly affected by the radar incidence angle and the topography of the study area.

**Figure 5:**
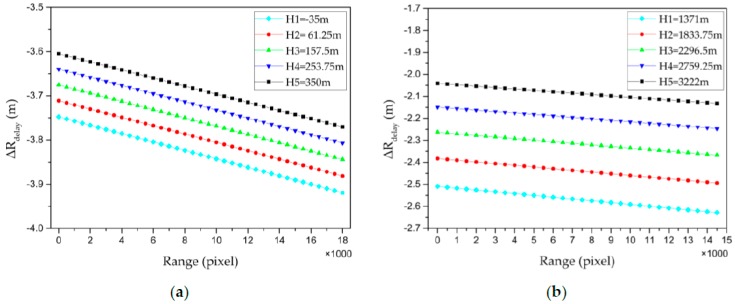
Trend of atmospheric delay correction value in the range direction: (**a**) plains and mountains area (ZJ-5851); (**b**) plateau area (ZY-7668).

**Figure 6:**
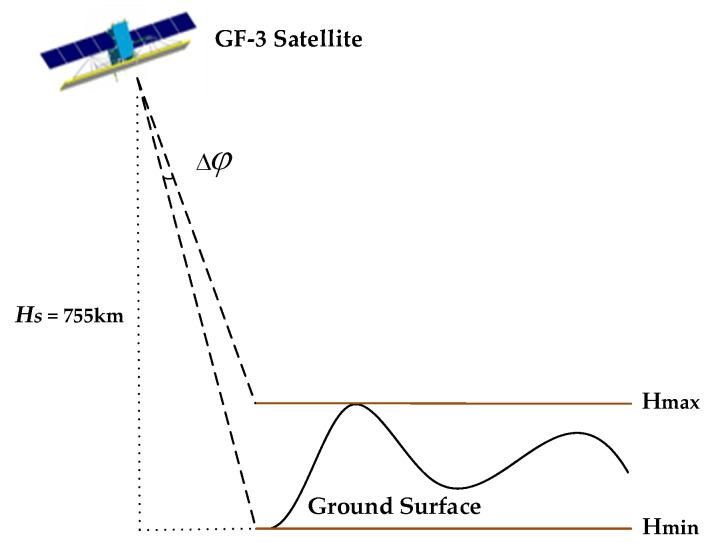
Variation of incident angle in the elevation direction in the study area.

**Figure 7:**
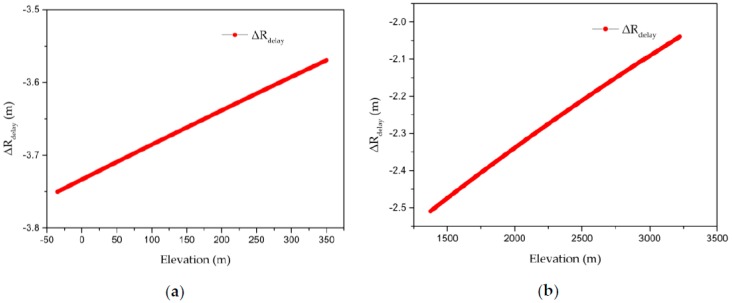
Atmospheric delay correction as a function of elevation: (**a**) plains and mountains area (ZJ-5851); (**b**) plateau area (ZY-7668).

To analyze the main influencing factors of the atmospheric propagation delay in the SAR image scenes further, we visualized the atmospheric propagation delay correction values of the virtual grid points calculated using plan 2, assuming this to be 0 pixels in the azimuth direction and sampling every 500 pixels in the range direction, with five elevation layers. The trend of the atmospheric delay correction value in the range direction is shown in Figure 5. To analyze the influence of the elevation on the atmospheric propagation delay, we increased the number of elevation layers of the virtual control grid to 300 separately in the plains and mountains area (ZJ-5851) and the plateau area (ZY-7668). The maximum variation *∆φ* of the corresponding incident angle at the same sampling point in each elevation layer in the experimental region is shown in Figure 6, where the orbital height of the satellite is known to be approximately 755 km. Here, *∆φ* was approximated using Equation (9), and was found to be less than 0.03° in the ZJ-5851 image coverage area and less than 0.15° in the ZY-7668 image coverage area. Thus, the variation of the incident angle in the elevation direction in the study area is negligible, and the same sampling point can be selected in each elevation layer to analyze the atmospheric propagation delay law as a function of elevation, as shown in Figure 7.

(9) $$\Delta \varphi <\frac{H_{max}}{H_{s}}\times \frac{180{}^{\circ}}{\pi },$$

where *H_s_* is the orbital height of the GF-3 satellite; *H_max_* and *H_min_* are the maximum and minimum elevations of the image coverage area, respectively.

Figure 5 shows that the atmospheric delay and incident angle are approximately linearly related at the same elevation level. The absolute value of the atmospheric propagation delay correction gradually increases as the range increases.

Some researchers have demonstrated that the elevation–atmospheric delay relationship is usually a linear or exponential function [20,21]. Figure 7a shows that in the plains and mountains area, the elevation–atmospheric delay relationship is approximately linear. Meanwhile, Figure 7b reveals that in the plateau region, the elevation–atmospheric delay relationship is also approximately linear.

According to the RPC model fitting parameter impact analysis results in Section 4.2.1, we established a control grid by setting the grid size to 500 pixels × 500 pixels and the number of elevation layers to five. The checkpoint was determined based on the centers of the four adjacent control points and two adjacent elevation layers, and the fitting accuracy of the RPC model was evaluated after correcting the atmospheric delay grid by grid. The results are presented in Table 3.

In general, as the plane error of the checkpoint does not exceed 5% of the pixels, we believe that the RPC model can be used instead of the RD model for photogrammetric processing of spaceborne SAR satellites. Table 3 shows that in the same scene, both the uncorrected atmospheric delay and atmospheric delay correction with plan 1 yield exactly the same RPC model fitting accuracy, and that the maximum plane RMSE of the checkpoint is 0.00356 pixels. For the atmospheric delay correction with plan 2, the fitting accuracy of the RPC model is very approximate, so both approaches achieve precision better than 1% of a pixel. This indicates that the RPC model still provides high accuracy for the RD model after correcting the atmospheric delay using two plans, in plain, mountainous, and plateau areas.

The reason for obtaining these experimental results can be confirmed by research that has shown that the third-order RPC model can better fit a function whose highest power is no more than 5 [22]. For a single-scene SAR image, plan 1 uses a unique atmospheric delay correction parameter to correct the atmospheric propagation delay at all virtual grid points, which is equivalent to adding the atmospheric propagation delay as a constant function to the slant range (R) term of the RD model. Therefore, the fitting accuracy of the RPC model is unchanged. Meanwhile, plan 2 corrects the atmospheric propagation delay at all virtual grid points with the correction parameters of the corresponding virtual grid points, which is equivalent to adding the atmospheric delay correction model to the R term with the elevation as the dependent variable. Figure 7a shows that the elevation–atmospheric delay function is approximately linear in the plains and mountains area, so it can be correctly fitted with a third-order RPC model. Meanwhile, Figure 7b shows that in the plateau area, the elevation–atmospheric delay function also shows an approximate linear relationship, which indicates that RPC model can not only fit complex terrain, but also fit the terrain-related atmospheric propagation delay.

#### 4.2.3. RPC Model Positioning Accuracy Verification 

From the perspective of RPC model application, we carried out RPC model positioning experiments to verify the effectiveness of the RPC model considering atmospheric propagation delay. Based on the experimental data of the GF-3 satellite and GCPs, geometric positioning verification experiments were performed for the four RPC models, and the results are shown in Table 4.

(1) Firstly, for the RPC model fitted using the original RD model without atmospheric delay correction, positioning accuracy verification was conducted;
(2) After correcting the systematic error in the slant range direction of the GF-3 SAR satellite, geometric positioning accuracy verification of the RPC model was performed. This systematic error was obtained by geometric calibration of the spaceborne SAR [23];
(3) After correcting the systematic error in the slant range direction of the GF-3 SAR satellite and correcting the atmospheric propagation delay error using plan 1, geometric positioning accuracy verification of the RPC model was performed;
(4) After correcting the systematic error in the slant range direction of the GF-3 SAR satellite and correcting the atmospheric propagation delay error using plan 2, geometric positioning accuracy verification of the RPC model was performed.

Table 4 shows that the positioning accuracy of the RPC model is between 21.241 m and 26.004 m without correction of system error and atmospheric delay error. After correcting the system error, the geometric positioning error of the RPC model was between 3.064 m and 5.529 m. After correcting the atmospheric delay error using plans 1 and 2, respectively, the maximum geometric positioning error of the RPC model was found to be 2.948 m and 2.957 m, and the positioning accuracy of a single scene image could be increased by 2.7 m, such as for ZJ-5492. Thus, it is effective to improve the geometric positioning accuracy of the RPC model by correcting the atmospheric propagation delay in the process of fitting the RD model to the RPC model. Furthermore, the positioning accuracy of the four RPC models in the azimuth direction is almost unchanged, which indicates that the slant range error caused by the atmospheric propagation delay mainly affects the positioning accuracy in the range direction.

**Figure 8:**
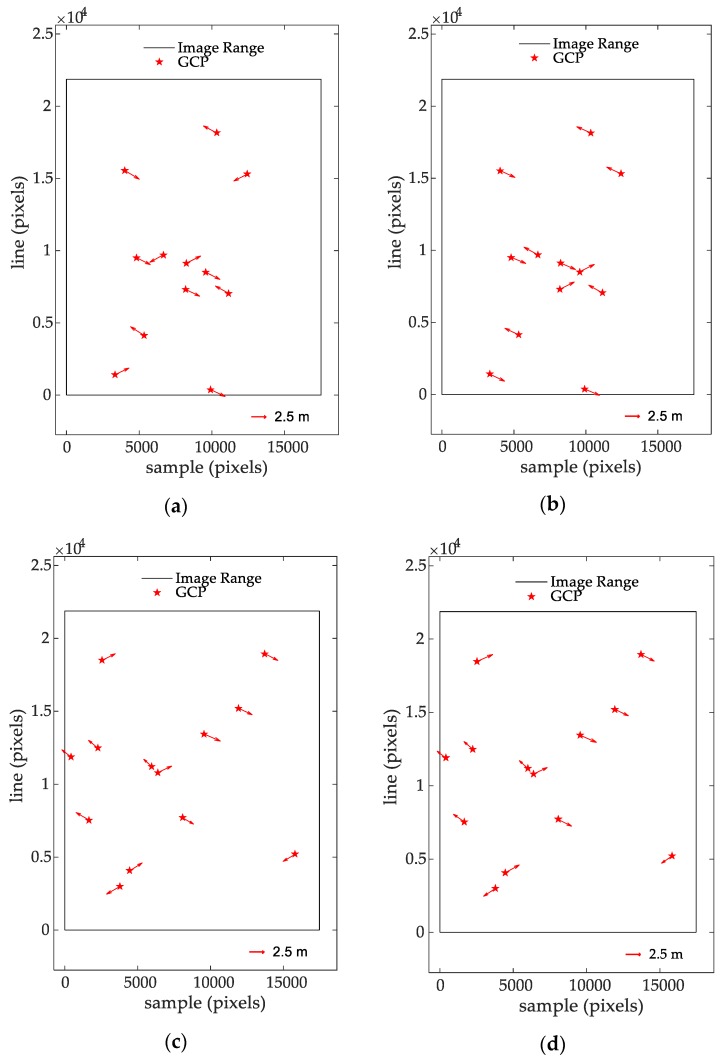
Positioning error distributions of all GCPs in one SAR image: (**a**) atmospheric delay corrected with plan 1 for image AP-6616; (**b**) atmospheric delay corrected with plan 2 for image AP-6616; (**c**) atmospheric delay corrected with plan 1 for image NM-3601; (**d**) atmospheric delay corrected with plan 2 for image NM-3601.

Figure 8 shows that the positioning error of all GCPs in the SAR image of a single scene is approximately 2.5 m after correcting the atmospheric propagation delay using plans 1 and 2 separately, and this remains relatively stable, which indicates that the geometric positioning results of the RPC model are reliable.

It is worth noting that although both plans are feasible, it is still difficult to produce a RPC model considering atmospheric delay directly and in real time in the SAR system, because sufficiently precise atmospheric models typically have latency. We may be able to choose a general model in which the atmospheric delay is a function of the SAR incidence angle and the elevation of a given point, in order to replace the precise atmospheric model [24]. This will be our next research direction.

## 5. Conclusions

The atmospheric delays of SAR satellite radar signals can reach several meters, which is non-negligible in high-precision geometric positioning. Thus, two methods of atmospheric delay correction during RPC fitting of an RD model were designed and tested in this study. Based on the GF-3 SAR satellite data, a global digital elevation model, global meteorological data, and ground control data from several regions in China, the feasibility of the two methods was verified by conducting RPC model fitting and positioning experiments. We found that the RPC model exhibited high substitution and positioning accuracies after using plan 1 or plan 2 to correct the atmospheric propagation delay, in both the plains and mountains area where the relative height difference is less than 1000 m, and in the plateau area where the relative height difference is more than 1000 m. Therefore, it is feasible to use plan 1 or plan 2 to correct the atmospheric delay error under any terrain conditions. Moreover, the geometric positioning accuracy of the RPC model after correcting the atmospheric delay was improved to better than 3 m.

## Figures and Tables

**Table 1:** GF-3 data information of the study area.

Imaging Mode	ID of Image	Imaging Region	Imaging Date	R*_r_*	R*_a_*	Number of GCPs
SL (1 m resolution)	CD-3076	Chengdou	8 January 2017	0.5621	0.3126	
	DF-6954	Dengfeng	7 August 2018	0.5621	0.3125	
	LZ-4905	Lanzhou	6 August 2018	0.5621	0.3443	
UFS (3 m resolution)	BC-1094	Beichen	18 September 2016	1.1242	1.7308	
	ZJ-8807	Zhanjiang	12 August 2017	1.1242	1.7327	
	SH-4753	Shanghai	27 January 2017	1.1242	1.7292	
FSI (5 m resolution)	AP-6616	Anping	19 February 2017	2.2484	2.8110	12
	AP-4130	Anping	5 May 2017	1.1242	2.6183	6
	ZJ-5851	Zhanjiang	25 January 2017	2.2484	2.8109	
	XN-3579	Xianning	11 January 2017	2.2484	2.8139	6
	NM-3601	Neimeng	11 January 2017	2.2484	2.8098	13
	NM-2661	Neimeng	28 July 2017	1.1242	2.5915	10
	ZY-7668	Zhangye	18 May 2019	2.2484	2.8252	5
FSII (10 m resolution)	CD-7465	Chengdou	3 October 2017	2.2484	4.7566	
	ZJ-5492	Zhanjiang	6 October 2017	2.2484	4.7710	8
	ZY-0316	Zhangye	22 January 2019	2.2484	4.7576	7

**Table 2:** Atmospheric delay correction value calculation results.

Topography	Image ID	Incidence Angle (°)		DEM (m)			ΔR_delay_ (m)	
		Near-Range	Far-Range	Minimum	Maximum	Mean	Plan 1	Plan 2
**Plains and mountains**	SH-4753	45.699	47.142	−30	36	0.00	−3.611	−3.678–−3.544
	AP-4130	26.182	29.657	−28	65	11.66	−2.825	−2.825–−2.712
	AP-6616	44.740	47.334	−27	72	23.54	−3.504	−3.623–−3.399
	BC-1094	19.759	22.199	−29	81	13.68	−2.813	−2.852–−2.768
	ZJ-8807	32.375	34.393	−30	169	24.05	−3.185	−3.258–−3.071
	ZJ-5851	46.510	48.971	−35	350	23.03	−3.795	−3.919–3.587
	XN-3579	44.777	47.362	−21	641	111.59	−3.393	−3.771–3.281
	CD-3076	26.160	27.019	426	975	695.23	−2.522	−2.613–−2.400
**Plateau**	ZJ-5492	31.291	38.155	−33	1271	22.52	−3.531	−3.702–2.824
	DF-6954	28.717	29.698	312	1470	446.73	−2.865	−2.943–−2.477
	NM-3601	44.746	47.334	976	1970	1052.78	−3.028	−3.516–−2.665
	NM-2661	23.865	27.674	952	2214	990.42	−2.561	−2.617–2.136
	ZY-7668	34.665	37.920	1371	3222	1498.87	−2.536	−2.636–2.041
	ZY-0316	30.894	37.837	1300	4769	2092.58	−2.28	−2.569–−1.568
	LZ-4905	41.035	41.745	1478	2150	1606.19	−3.012	−3.095–−2.780
	CD-7465	31.294	37.718	390	4931	810.42	−3.111	−3.212–1.521

**Table 3:** Fitting accuracy of the RPC model. Note: Max. = maximum; RMSE = root mean square error.

Topography	Image ID	Compensation Method	Control Point Error (Pixels)						Check Point Error (Pixels)					
			Sample		Line		2-D		Sample		Line		2-D	
			Max.	RMSE	Max.	RMSE	Max.	RMSE	Max.	RMSE	Max.	RMSE	Max.	RMSE
**Plains and Mountains**	SH-4753	Uncorrected/Plan 1	0.00041	0.00013	0.00013	0.00007	0.00041	0.00015	0.00029	0.00012	0.00015	0.00007	0.0003	0.00014
		Plan 2	0.00041	0.00013	0.00013	0.00007	0.00041	0.00015	0.00029	0.00012	0.00015	0.00007	0.0003	0.00014
	AP-4130	Uncorrected/Plan 1	0.0122	0.00364	0.0032	0.0012	0.01245	0.00383	0.00933	0.00338	0.00219	0.00111	0.0095	0.00356
		Plan 2	0.01243	0.00366	0.0032	0.0012	0.01268	0.00385	0.00944	0.0034	0.00219	0.00111	0.00961	0.00357
	AP-6616	Uncorrected/Plan 1	0.00172	0.00059	0.00009	0.00004	0.00172	0.00059	0.00126	0.00055	0.00012	0.00005	0.00126	0.00055
		Plan 2	0.00171	0.00059	0.00009	0.00004	0.00171	0.0006	0.00127	0.00055	0.00012	0.00005	0.00127	0.00055
	BC-1094	Uncorrected/Plan 1	0.00074	0.00025	0.00012	0.00007	0.00075	0.00026	0.00053	0.00022	0.00016	0.00007	0.00055	0.00024
		Plan 2	0.00073	0.00026	0.00012	0.00007	0.00074	0.00027	0.00053	0.00023	0.00016	0.00007	0.00055	0.00025
	ZJ-8807	Uncorrected/Plan 1	0.00077	0.00024	0.0012	0.00047	0.00132	0.00052	0.00059	0.00022	0.00077	0.00043	0.00084	0.00048
		Plan 2	0.00078	0.00024	0.0012	0.00047	0.00132	0.00052	0.00059	0.00022	0.00077	0.00043	0.00084	0.00048
	ZJ-5851	Uncorrected/Plan 1	0.00276	0.0009	0.00164	0.00063	0.00318	0.0011	0.00203	0.00083	0.001	0.00058	0.00218	0.00102
		Plan 2	0.00276	0.0009	0.00164	0.00063	0.00316	0.0011	0.00203	0.00083	0.001	0.00058	0.00218	0.00102
	XN-3579	Uncorrected/Plan 1	0.00084	0.0002	0.00031	0.00007	0.00089	0.00021	0.00056	0.00018	0.0002	0.00007	0.00059	0.0002
		Plan 2	0.00083	0.00019	0.00031	0.00007	0.00088	0.00021	0.00054	0.00018	0.0002	0.00007	0.00057	0.00019
	CD-3076	Uncorrected/Plan 1	0.00015	0.00005	0.00066	0.00032	0.00066	0.00033	0.00012	0.00005	0.00093	0.00046	0.00093	0.00046
		Plan 2	0.00015	0.00005	0.00066	0.00032	0.00066	0.00033	0.00012	0.00005	0.00093	0.00046	0.00093	0.00046
**Plateau**	ZJ-5492	Uncorrected/Plan 1	0.00839	0.00207	0.00058	0.00014	0.00839	0.00207	0.00658	0.00192	0.00044	0.00013	0.00658	0.00193
		Plan 2	0.00843	0.00209	0.00058	0.00014	0.00843	0.00209	0.0066	0.00194	0.00044	0.00013	0.0066	0.00194
	DF-6954	Uncorrected/Plan 1	0.00134	0.00046	0.00079	0.00039	0.00146	0.00061	0.00112	0.00044	0.00119	0.00043	0.00158	0.00061
		Plan 2	0.00134	0.00047	0.00079	0.00039	0.00147	0.00061	0.00112	0.00044	0.00119	0.00043	0.00158	0.00062
	NM-3601	Uncorrected/Plan 1	0.00072	0.00018	0.00026	0.00011	0.00074	0.00021	0.00054	0.00017	0.00024	0.0001	0.00055	0.0002
		Plan 2	0.00075	0.00019	0.00026	0.00011	0.00077	0.00022	0.00057	0.00017	0.00024	0.0001	0.00059	0.0002
	NM-2661	Uncorrected/Plan 1	0.00832	0.00179	0.00106	0.00041	0.00839	0.00184	0.00555	0.00165	0.00078	0.00038	0.00561	0.00169
		Plan 2	0.00833	0.00179	0.00106	0.00041	0.00839	0.00184	0.00553	0.00165	0.00078	0.00038	0.00559	0.00169
	ZY-7668	Uncorrected/Plan 1	0.00111	0.00025	0.00167	0.00067	0.00185	0.00072	0.00073	0.00023	0.00113	0.00065	0.00134	0.00069
		Plan 2	0.00123	0.00026	0.00167	0.00071	0.00203	0.00075	0.00074	0.00023	0.00113	0.00065	0.00134	0.00069
	ZY-0316	Uncorrected/Plan 1	0.0106	0.00216	0.00141	0.00033	0.01061	0.00219	0.00756	0.00201	0.00094	0.00031	0.00756	0.00203
		Plan 2	0.0106	0.00215	0.00142	0.00033	0.01061	0.00217	0.00752	0.00199	0.00094	0.00031	0.00752	0.00202
	LZ-4905	Uncorrected/Plan 1	0.0018	0.00067	0.00133	0.00051	0.00224	0.00085	0.00149	0.00064	0.00158	0.00053	0.00216	0.00083
		Plan 2	0.00181	0.00067	0.00133	0.00051	0.00222	0.00085	0.0015	0.00064	0.00158	0.00053	0.00216	0.00083
	CD-7465	Uncorrected/Plan 1	0.01	0.002	0.0067	0.00241	0.01113	0.00371	0.00826	0.00261	0.00469	0.00223	0.00897	0.00344
		Plan 2	0.01	0.002	0.0067	0.00241	0.01112	0.0037	0.00827	0.00261	0.00469	0.00223	0.00899	0.00344

**Table 4:** Geometric positioning accuracies of the four RPC models.

Image ID	Correction Method											
	Uncorrected			Correct System Error			Plan 1			Plan 2		
	Sample	Line	2-D	Sample	Line	2-D	Sample	Line	2-D	Sample	Line	2-D
AP-6616	22.858	1.380	22.900	4.021	1.380	4.409	2.588	1.380	2.934	2.599	1.380	2.944
AP-4130	21.619	1.000	21.642	2.782	1.000	3.064	1.878	1.000	2.129	1.871	1.000	2.123
XN-3579	22.397	1.457	22.446	3.561	1.457	4.004	2.382	1.457	2.801	2.380	1.457	2.792
NM-3601	22.460	0.118	22.496	3.623	0.117	3.941	2.110	0.117	2.459	0.510	0.118	2.486
NM-2661	21.207	1.188	21.241	2.730	1.188	3.075	1.603	1.188	2.021	1.602	1.188	2.021
ZY-7668	23.198	1.372	23.238	4.360	1.373	4.571	1.447	1.372	1.994	1.388	1.372	1.952
ZJ-5492	25.899	2.333	26.004	5.013	2.333	5.529	1.600	2.332	2.828	1.885	2.333	2.999
ZY-0316	24.983	1.001	25.003	4.096	1.000	4.217	1.184	1.000	1.550	1.166	1.000	1.536
